# When Is Higher Neuroticism Protective Against Death? Findings From UK Biobank

**DOI:** 10.1177/0956797617709813

**Published:** 2017-07-13

**Authors:** Catharine R. Gale, Iva Čukić, G. David Batty, Andrew M. McIntosh, Alexander Weiss, Ian J. Deary

**Affiliations:** 1 Centre for Cognitive Ageing and Cognitive Epidemiology, Department of Psychology, University of Edinburgh; 2 MRC Lifecourse Epidemiology Unit, University of Southampton; 3 Department of Psychology, University of Edinburgh; 4 Department of Epidemiology & Public Health, University College London; 5 Division of Psychiatry, University of Edinburgh

**Keywords:** neuroticism, self-rated health, mortality, cohort study

## Abstract

We examined the association between neuroticism and mortality in a sample of 321,456 people from UK Biobank and explored the influence of self-rated health on this relationship. After adjustment for age and sex, a 1-*SD* increment in neuroticism was associated with a 6% increase in all-cause mortality (hazard ratio = 1.06, 95% confidence interval = [1.03, 1.09]). After adjustment for other covariates, and, in particular, self-rated health, higher neuroticism was associated with an 8% reduction in all-cause mortality (hazard ratio = 0.92, 95% confidence interval = [0.89, 0.95]), as well as with reductions in mortality from cancer, cardiovascular disease, and respiratory disease, but not external causes. Further analyses revealed that higher neuroticism was associated with lower mortality only in those people with fair or poor self-rated health, and that higher scores on a facet of neuroticism related to worry and vulnerability were associated with lower mortality. Research into associations between personality facets and mortality may elucidate mechanisms underlying neuroticism’s covert protection against death.

People with higher levels of the personality trait neuroticism—the tendency to experience negative emotions—are more likely to rate their health as poor (Goodwin & Engstrom, 2002; Jorm et al., 1993) and to report somatic complaints (Costa & McCrae, 1987; Neeleman, Bijl, & Ormel, 2004). They are also at increased risk of common mental disorders (Kotov, Gamez, Schmidt, & Watson, 2010; Lonnqvist et al., 2009; Malouff, Thorsteinsson, & Schutte, 2005). Given the evidence indicating that people with higher levels of psychological distress are more likely to die sooner than people with lower levels (Gale et al., 2012; Russ et al., 2012), one might expect that higher neuroticism would be associated with increased mortality, but findings regarding this prediction are inconsistent.

Whereas some studies have found associations between higher neuroticism and increased mortality (Shipley, Weiss, Der, Taylor, & Deary, 2007; Weiss, Gale, Batty, & Deary, 2009), others have found no link (Almada et al., 1991; Costa, Weiss, Duberstein, Friedman, & Siegler, 2014; Iwasa et al., 2008; Jokela et al., 2013). In fact, a few studies have found that neuroticism might protect against mortality (Korten et al., 1999; Ploubidis & Grundy, 2009; Weiss & Costa, 2005; Weiss, Gale, Batty, & Deary, 2013). One explanation for such a protective effect might be that some variable moderates the relationship between neuroticism and mortality. For example, there is some evidence that when high neuroticism is accompanied by high conscientiousness, it may have benefits for health, as indicated by lower levels of inflammatory biomarkers (Turiano, Mroczek, Moynihan, & Chapman, 2013), less smoking after the onset of disease (Weston & Jackson, 2015), and lower mortality—albeit in women only (Friedman, Kern, & Reynolds, 2010). The idea that higher neuroticism might have health advantages in certain circumstances—the concept of “healthy neuroticism”—was first put forward by Friedman (2000), who suggested that some people who are high in neuroticism may be vigilant about their health and seek medical advice more readily than other people do.

Another plausible moderator of the neuroticism-mortality relationship may be self-rated health, which predicts mortality independently of objectively measured health (Benyamini & Idler, 1999; Ganna & Ingelsson, 2015; Idler & Benyamini, 1997). People who are higher in neuroticism are more likely to rate their health as poor (Chapman, Duberstein, & Lyness, 2007; Goodwin & Engstrom, 2002). Indications that self-rated health interacts with neuroticism to affect mortality risk come from studies in which higher neuroticism was associated with lower mortality when effect estimates were adjusted for self-rated health (Korten et al., 1999; Weiss & Costa, 2005). Korten et al. (1999) reported that this association was not apparent in a univariate analysis, but they discussed the role of neuroticism solely as a confounder of the relationship between self-rated health and mortality rather than considering why it should become protective after adjustment. In addition to possibly moderating the association between neuroticism and mortality, self-rated health might act as a mediator. Longitudinal evidence shows that higher neuroticism is associated with faster decline in self-rated health, which might contribute to mortality risk (Löckenhoff, Terracciano, Ferrucci, & Costa, 2012). A related possibility is highlighted by a study by Ploubidis and Grundy (2009), which showed that neuroticism had both an indirect and a direct relationship with mortality risk. Higher neuroticism was indirectly related to greater risk via mediators, including self-rated health, but, in women only, higher neuroticism was also directly related to reduced risk.

We used data from UK Biobank to investigate the association between neuroticism and mortality. Our aim was to investigate whether and how self-rated health influences the relationship between neuroticism and risk of death from all causes, cancer, cardiovascular disease, respiratory disease, and external causes.

## Method

### Participants

The participants in this study took part in the baseline survey of UK Biobank (Sudlow et al., 2015; see also UK Biobank’s Web site: http://www.ukbiobank.ac.uk), a resource established for identifying determinants of disease in middle-aged and older people. Between 2006 and 2010, 502,655 community-dwelling people who were ages 37 to 73 years and living in the United Kingdom were recruited to the study. UK Biobank received ethical approval from the North West Multi-Centre Research Ethics Committee (Reference 11/NW/0382).

### Measures

#### Neuroticism

Participants completed the 12-item Neuroticism scale of the Eysenck Personality Questionnaire-Revised (EPQ-R) Short Form (Eysenck, Eysenck, & Barrett, 1985). Response options were “true,” “false,” “do not know,” and “prefer not to answer”; the latter two responses were coded as missing data. We used the summed score for our main analyses. The EPQ-R Short Form has been concurrently validated in older people using the Emotional Stability scale of the International Personality Item Pool (*r* = −.84) and the Neuroticism domain of the NEO Five-Factor Inventory (*r* = .85; Gow, Whiteman, Pattie, & Deary, 2005).

#### Self-rated health

Participants were asked, “In general how would you rate your overall health?” Responses were coded as “excellent,” “good,” “fair,” or “poor.”

#### Other covariates

In addition to age, sex, and self-rated health, we chose to include various health behaviors, physical attributes, cognitive function, diagnosed disease, and socioeconomic position as covariates on the grounds that they might mediate or confound the relationships between neuroticism and mortality. All the covariates were assessed along with neuroticism during the baseline survey. It was therefore not possible to be certain about the temporal ordering of all of the covariates.

Health behaviors included were smoking status (never smoked, ex-smoker, current smoker), frequency of alcohol intake (never, on special occasions only, one to three times a month, once or twice a week, three or four times a week, daily, or almost daily), consumption of five or more portions of fruit and vegetables per day (yes or no), and number of types of physical activity performed in the last 4 weeks. The categories of physical activity were walking for pleasure, heavy do-it-yourself activities (e.g., weeding, lawn mowing, carpentry, digging), light do-it-yourself activities (e.g., pruning, watering the lawn), strenuous sports, and other exercise.

Physical attributes included body mass index (BMI), systolic blood pressure, forced expiratory volume in 1 s (FEV1), and grip strength, all of which were measured during a visit to a UK Biobank Assessment Centre. Body mass index (kilograms/meter^2^) was calculated from height and weight. Systolic blood pressure was measured with an automated Omron device (www.omronhealthcare.com). FEV1 was measured using a Vitalograph (www.vitalograph.com) Pneumotrac 6800. Grip strength of each hand was measured using a Jamar J00105 hydraulic hand dynamometer (Lafayette Instrument, Lafayette, IN); the maximum value was used in our analyses.

Our measure of cognitive function was reaction time. Reaction time and scores on other measures of processing speed are moderately highly correlated with intelligence; people with higher intelligence tend to process information more quickly (Deary, Der, & Ford, 2001). Reaction time was measured using a go/no-go “Snap” game. Via a computer screen, participants were presented with two cards with symbols on them. Participants were instructed that if the cards were identical, they should push a button as quickly as possible using their dominant hand; otherwise, they should not respond. Twelve pairs of cards were shown. The first five pairs were used as a practice. Of the remaining seven pairs, four contained identical cards. The score for reaction time was the mean time in milliseconds before a participant pressed the button when one of these four pairs was presented. Internal consistency of the four test trials was high (Cronbach’s α = .85).

Diagnosed disease was assessed via self-report. Participants indicated whether they had been diagnosed by a physician with vascular or heart problems, diabetes, cancer, chronic bronchitis or emphysema, asthma, deep vein thrombosis, or pulmonary embolism.

Socioeconomic position was assessed using each participant’s highest educational qualification and Townsend deprivation score (Townsend, Phillimore, & Beattie, 1988). The latter score was based on census data on unemployment, car and house ownership, and overcrowding for the participant’s postcode of residence.

#### Mortality

We used death certificates from the National Health Service Central Registry to identify the causes of death for those participants who died during the study period, which ended June 12, 2015. In addition to examining mortality from all causes, we looked at cause-specific mortality, using the International Statistical Classification of Diseases, 10th revision (World Health Organisation, 1992), to categorize deaths as due to cardiovascular disease (codes I20-5, I50, I60-70, I73, I74), cancer (codes C00-C97), respiratory disease (codes J00-J99), or external causes (codes V01-Y99). Any mention of any of these causes on a death certificate was counted as death from that cause. The mean follow-up time was 6.25 years.

### Statistical analysis

Having checked that the assumption of proportional hazards was met, we used Cox proportional-hazards regressions to examine all-cause and cause-specific mortality per 1-*SD* increment in neuroticism. Survival time in days was calculated from date of attendance at the Assessment Centre to date of death or June 12, 2015, whichever occurred first. In examining associations between neuroticism and all-cause and cause-specific mortality, we initially adjusted for age and sex, and then further adjusted for health behaviors, physical attributes, reaction time, diagnosed disease, and socioeconomic position; finally, we adjusted for self-rated health. We estimated the impact on the hazard ratio (HR) of adjusting for individual covariates using the following formula described by Batty, Der, Macintyre, and Deary (2006):

$$\begin{array}{l} ([\mathrm{HR adjusted for age and sex}-1]-\\ {}[\mathrm{HR adjusted for age},\mathrm{sex},\mathrm{and third covariate}-1])/\\ (\mathrm{HR adjusted for age and sex}-1)\times 100. \end{array}$$

We then examined whether relationships between neuroticism and all-cause and cause-specific mortality were moderated by levels of self-rated health by including interaction terms in age- and sex-adjusted models and testing whether the interactions were statistically significant. We also examined the relationships between neuroticism and all-cause and cause-specific mortality at each level of self-rated health with adjustments for the other covariates.

Neuroticism has a hierarchical structure, as do other personality factors (Costa & McCrae, 1995); items define lower-order facets, which, in turn, define the factor. Therefore, we examined whether any neuroticism facet or facets uniquely predicted mortality risk, or whether the association between neuroticism and mortality risk was attributable to the common variance. To do so, we first ran an exploratory structural equation model with an oblique bifactor Geomin rotation (Jennrich & Bentler, 2011, 2012) in Mplus Version 7.4 (Muthén & Muthén, 1998–2015) to extract a general Neuroticism factor and two facets that were orthogonal to the general factor but correlated with each other. Next, we entered the general Neuroticism factor score and the facet scores, simultaneously, in further Cox models that were like those described earlier.

We carried out multiple tests of statistical significance. To reduce the likelihood of false positive results, we adjusted the *p* values for the false discovery rate (FDR; Benjamini, Drai, Elmer, Kafkafi, & Golani, 2001). We report results with and without this correction. In view of the very large sample size, only *p* values below .001 were considered statistically significant.

Our analytical sample included 321,456 participants (64% of the 502,655 people recruited to UK Biobank) who had complete data on neuroticism, self-rated health, and the other covariates at baseline and on mortality during the follow-up.

## Results

Table 1 shows the baseline characteristics of the study participants, separately for those who did and did not die during the follow-up period. In this very large sample with 4,497 deaths, most of our covariates were significantly associated with survival: Death in the follow-up period was associated with older age, being male, being a current smoker, drinking alcohol daily or almost daily, engaging in fewer types of exercise, eating fewer than five portions of fruits and vegetables per day, higher BMI, higher systolic blood pressure, lower FEV1, slower reaction time, having diagnoses of various physical diseases, not having a university degree, living in an area of greater social deprivation, and poorer self-rated health (*p*s < .0001).

**Table 1.** Comparison of the Baseline Characteristics of the Study Participants Who Did and Did Not Survive Until the End of the Follow-Up Period (*N* = 321,456)

Characteristic	Died during follow-up		Group comparison: *p* value
	Yes (*n* = 4,497)	No (*n* = 316,959)	
Age (years)	*M* = 61.0 (*SD* = 6.76)	*M* = 56.1 (*SD* = 8.06)	< .0001
Female	*n* = 1,784 (39.7%)	*n* = 171,943 (54.3%)	< .0001
Neuroticism	*M* = 3.89 (*SD* = 3.23)	*M* = 4.06 (*SD* = 3.24)	.0005
Fair or poor self-rated health	*n* = 1,829 (40.7%)	*n* = 69,144 (21.8%)	< .0001
Current smoker	*n* = 801 (17.8%)	*n* = 30,471 (9.61%)	< .0001
< 5 portions of fruits and vegetables per day	*n* = 1,599 (35.6%)	*n* = 123,748 (39.0%)	< .0001
Alcohol daily or almost daily	*n* = 1,119 (24.5%)	*n* = 68,188 (21.2%)	< .0001
Types of physical activity in the last 4 weeks	*M* = 1.96 (*SD* = 1.17)	*M* = 2.31 (*SD* = 1.16)	< .0001
BMI (kg/m^2^)	*M* = 28.0 (*SD* = 5.24)	*M* = 27.3 (*SD* = 4.66)	< .0001
Systolic blood pressure (mm Hg)	*M* = 139.2 (*SD* = 19.9)	*M* = 135.3 (*SD* = 18.4)	< .0001
Grip strength (kg)	*M* = 32.7 (*SD* = 11.0)	*M* = 33.0 (*SD* = 11.3)	.022
FEV1 (liters)	*M* = 2.62 (*SD* = 0.84)	*M* = 2.85 (*SD* = 0.80)	< .0001
Reaction time (ms)	*M* = 587.0 (*SD* = 126.8)	*M* = 553.7 (*SD* = 113.1)	< .0001
Vascular or heart problems	*n* = 1,946 (43.3%)	*n* = 87,348 (27.6%)	< .0001
Diabetes	*n* = 507 (11.3%)	*n* = 14,432 (4.55%)	< .0001
Asthma	*n* = 441 (9.81%)	*n* = 34,428 (10.9%)	.024
Chronic lung disease	*n* = 175 (3.89%)	*n* = 3,547 (1.12%)	< .0001
Cancer	*n* = 1,141 (25.4%)	*n* = 22,655 (7.15%)	< .0001
Deep vein thrombosis	*n* = 162 (3.60%)	*n* = 5,644 (1.78%)	< .0001
Pulmonary embolism	*n* = 67 (1.49%)	*n* = 2,181 (0.69%)	< .0001
University degree	*n* = 1,169 (26.0%)	*n* = 109,818 (34.7%)	< .0001
Townsend index	*Mdn* = −1.93 (IQR = −3.55 to 1.01)	*Mdn* = −2.29 (IQR −3.70 to 0.19)	< .0001

Note: BMI = body mass index; FEV1 = forced expiratory volume in 1 s; IQR = interquartile range.

### Neuroticism and mortality

Mean neuroticism scores were lower among participants who died during the follow-up period than among those who survived. This difference arose because men tended to score lower in neuroticism and have higher mortality: When men and women were analyzed separately, there was no difference in mean neuroticism between participants who survived and participants who died: mean scores were 3.54 (*SD* = 3.17) and 3.54 (*SD* = 3.15), respectively, for men (*p* = .987) and 4.50 (*SD* = 3.23) and 4.43 (*SD* = 3.28) for women (*p* = .330).

People who were higher in neuroticism rated their health as poorer; the rank-order correlation between neuroticism and self-rated health (based on four categories) was significant, *r*_S_ = .23, *p* < .0001. Neuroticism scores tended to be lower with increasing age, *r* = −.10, *p* < .0001.

Table 2 shows HRs and 95% confidence intervals (CIs) for all-cause and cause-specific mortality per 1-*SD* increment in neuroticism. In the age- and sex-adjusted analysis, all-cause mortality was higher in study participants with higher levels of neuroticism, HR = 1.06, 95% CI = [1.03, 1.09]. In additional models, we made further separate adjustments for the other covariates to gauge the impact of each on the association between neuroticism and mortality. Adjustment for health behaviors had the strongest attenuating effect on the association, reducing it by 100%. Adjustment for physical attributes, socioeconomic position, and existing illness each attenuated the association by 50%. Adjustment for reaction time attenuated the association only by 17%. Following adjustment for self-rated health, the association between neuroticism and mortality reversed direction, such that higher neuroticism was significantly linked with lower mortality, HR = 0.93, 95% CI = [0.90, 0.96]. The size of this reversed effect was little changed when the model simultaneously adjusted for all the covariates: A 1-*SD* increase in neuroticism was associated with an 8% reduction in mortality risk, HR = 0.92, 95% CI = [0.89, 0.95]. After FDR correction, these latter two associations remained significant, *p* < .001.

**Table 2.** Hazard Ratios (HRs) for All-Cause and Cause-Specific Mortality per 1-*SD* Increment in Neuroticism (*N* = 321,456)

Adjustment	Death from all causes (*n* = 4,497)		Death from cancer (*n* = 2,912)		Death from cardiovascular disease (*n* = 925)		Death from respiratory disease (*n* = 688)		Death from external causes (*n* = 422)	
	HR	*p, p*_FDR_	HR	*p, p*_FDR_	HR	*p, p*_FDR_	HR	*p*, *p*_FDR_	HR	*p, p*_FDR_
Age, sex	1.06 [1.03, 1.09]	< .001, < .001	1.00 [0.97, 1.04]	.776, .887	1.08 [1.01, 1.15]	.027, .072	1.11 [1.03, 1.20]	.007, .028	1.20 [1.09, 1.32]	< .001, < .001
Age, sex, and health behaviors	1.00 [0.98, 1.04]	.642, .660	0.96 [0.93, 1.00]	.065, .173	1.00 [0.94, 1.07]	.950, .975	1.03 [0.95, 1.11]	.491, .650	1.14 [1.04, 1.26]	.005, .003
Age, sex, and physical attributes	1.03 [1.00, 1.06]	.049, .057	0.99 [0.95, 1.03]	.629, .841	1.03 [0.97, 1.10]	.337, .449	1.05 [0.97, 1.13]	.225, .257	1.17 [1.06, 1.28]	.001, .003
Age, sex, and reaction time	1.05 [1.02, 1.08]	.001, .002	1.00 [0.96, 1.04]	.907, .907	1.07 [1.00, 1.14]	.054, .108	1.10 [1.01, 1.18]	.017, .034	1.19 [1.08, 1.30]	< .001, .001
Age, sex, and SEP	1.03 [1.00, 1.06]	.050, .057	0.99 [0.95, 1.02]	.631, .841	1.03 [0.97, 1.11]	.563, .643	1.06 [0.98, 1.15]	.203, .257	1.16 [1.06, 1.28]	.002, .003
Age, sex, and existing illness	1.03 [1.00, 1.06]	.033, .053	0.99 [0.95, 1.03]	.508, .841	1.02 [0.96, 1.09]	.314, .449	1.05 [0.97, 1.13]	.127, .203	1.16 [1.06, 1.27]	002, .003
Age, sex, and self-rated health	0.93 [0.90, 0.96]	< .001, < .001	0.90 [0.87, 0.94]	< .001, < .001	0.91 [0.85, 0.98]	.046, .024	0.90 [0.83, 0.98]	.011, .029	1.07 [0.97, 1.18]	.148, .169
All covariates	0.92 [0.89, 0.95]	< .001, < .001	0.90 [0.86, 0.93]	< .001, < .001	0.89 [0.83, 0.95]	.001, .008	0.87 [0.80, 0.94]	.001, .008	1.04 [0.95, 1.15]	.383, .383

Note: Effect estimates were first adjusted for age and sex only and then further adjusted separately for other covariates at baseline: health behaviors (smoking status, frequency of alcohol intake, number of types of exercise engaged in, and daily consumption of fruits and vegetables), physical attributes (body mass index, forced expiratory volume in 1 s, systolic blood pressure, and grip strength), reaction time, existing illness (diagnosis of vascular or heart problems, diabetes, cancer, asthma, chronic lung disease, deep vein thrombosis, or pulmonary embolism at baseline), socioeconomic position (SEP; Townsend index score and highest educational qualification), and self-rated health. Finally, estimates were adjusted for all the covariates simultaneously. Values inside brackets are 95% confidence intervals. *p* = uncorrected *p* value; *p*_FDR_ = *p* value corrected for the false discovery rate.

Cancer was the most common cause of death in the study sample. There was no significant association between neuroticism and risk of death from cancer in the age- and sex-adjusted analysis. Further separate adjustment for health behaviors, physical attributes, reaction time, socioeconomic position, and existing illness had little effect on the association between neuroticism and cancer mortality, and it remained nonsignificant. Following adjustment for self-rated health, higher neuroticism became significantly linked with lower risk of death from cancer, HR = 0.90, 95% CI = [0.87, 0.94]. The size of this effect was unchanged by simultaneous adjustment for all covariates: A 1-*SD* increase in neuroticism was associated with a 10% reduction in risk, HR = 0.90, 95% CI = [0.86, 0.93]. After FDR correction, these latter two associations remained significant, *p* < .001.

People who were higher in neuroticism tended to have an increased risk of death from cardiovascular disease and respiratory disease in the age- and sex-adjusted analysis, although neither of these associations was statistically significant either before or after FDR correction. As with all-cause mortality, we observed a reversal of the association between neuroticism and mortality specifically after adjustment for self-rated health. After this adjustment, neuroticism was associated with a reduced risk of death from both cardiovascular disease, HR = 0.91, 95% CI = [0.85, 0.98], and respiratory disease, HR = 0.90, 95% CI = [0.83, 0.98]. Neither of these associations was significant at *p* < .001.The effect sizes increased slightly after simultaneous adjustment for all the covariates: A 1-*SD* increment in neuroticism was associated with a reduction in risk of 11% for mortality due to cardiovascular disease, HR = 0.89, 95% CI = [0.83, 0.95], and with a reduction in risk of 13% for mortality due to respiratory disease, HR = 0.87, 95% CI = [0.80, 0.94]. These latter models (both *p*s = .001) did not meet our criterion for significance before FDR correction; after FDR correction, they had *p* values of .008.

The results for death from external causes showed a different pattern. Higher neuroticism was associated with increased risk of death from external causes in the age- and sex-adjusted analysis, and this association was significant both before and after FDR correction. Separate adjustments for health behaviors, physical attributes, reaction time, socioeconomic position, and existing illness each attenuated this association, by between 30% (health behaviors) and 5% (reaction time). Separate adjustment for self-rated health attenuated the association by 65%, and rendered it nonsignificant, *p* = .148. Simultaneous adjustment for all the covariates attenuated the relationship still further.

In summary, age- and sex-adjusted analyses showed that higher neuroticism was associated with a slight increase in all-cause mortality. However, after adjustment for self-rated health, higher neuroticism was associated with reduced mortality from all causes and cancer (both *p*s < .001) and with nonsignificant reductions in mortality from cardiovascular disease and respiratory disease (both *p*s = .008 after FDR correction). Higher neuroticism was associated with increased mortality from external causes, but this association was no longer significant after adjustment for self-rated health and other covariates.

### The neuroticism-mortality association by level of self-rated health

We next examined whether the associations between neuroticism and mortality from all causes, cancer, cardiovascular disease, respiratory disease, and external causes varied according to level of self-rated health. Tests of the interaction between neuroticism and self-rated health met our imposed level of significance (*p* < .001) in the case of mortality from cancer (*p* = .0007), but not in the case of mortality from all causes *(p* = .003), cardiovascular disease (*p* = .806), respiratory disease (*p* = .362), or external causes (*p* = .734). For mortality from all causes and mortality from cancer, we compared the models that included the interaction with the models that did not include it. We found that the model with the interaction fitted the data better than the model without the interaction only in the case of mortality from cancer. The likelihood ratio test statistics (distributed as a chi-square, *df* = 3) were 13.81 for all-cause mortality (*p* = .003) and 17.14 for cancer-related mortality (*p* = .0007). Likelihood ratio tests are sensitive to sample size, so these results should be viewed with caution.

Next, we carried out exploratory analyses in which we examined the associations between neuroticism and mortality, stratifying by self-rated health. Table 3 shows the HRs and 95% CIs for death from all causes and specific causes per 1-*SD* increment in neuroticism for each level of self-rated health. (Results for mortality from all causes, cardiovascular disease, respiratory disease, and external causes are included in the table to provide full results, even though the relationship between neuroticism and these causes of death did not vary by level of self-rated health.) For all causes of death, neuroticism was significantly protective against mortality in participants who rated their health as fair or poor (*p* < .001), but not in those who rated their health as excellent or good. The age- and sex-adjusted HR was 0.89, 95% CI = [0.83, 0.94], for participants who rated their health as fair and 0.83, 95% CI = [0.76, 0.90], for participants who rated their health as poor. After adjustment for all the covariates, the corresponding HRs were 0.89, 95% CI = [0.83, 0.94], and 0.86, 95% CI = [0.79, 0.94]. Both associations were statistically significant at conventional levels, but only the association among participants with fair self-rated health met our more stringent criterion for significance (*p* < .001) after FDR correction. For cancer-related mortality, too, neuroticism was significantly protective in participants who rated their health as fair or poor. The age- and sex-adjusted HR for death from cancer per 1-*SD* increment in neuroticism was 0.87, 95% CI = [0.80, 0.94], for those who rated their health as fair and 0.73, 95% CI = [0.65, 0.82], for those who rated their health as poor. Further adjustment for all the covariates had little or no attenuating effects on these associations: The multivariable-adjusted HRs were 0.87, 95% CI = [0.81, 0.94], for participants who rated their health as fair and 0.80, 95% CI = [0.71, 0.90], for those who rated their health as poor, and both associations remained statistically significant after FDR correction.

**Table 3.** Hazard Ratios (HRs) for All-Cause and Cause-Specific Mortality per 1-*SD* Increment in Neuroticism, Stratified by Level of Self-Rated Health (*N* = 321,456)

Cause of death and adjustments	Excellent health (*n* = 59,305)			Good health (*n* = 191,178)			Fair health (*n* = 60,095)			Poor health (*n* = 10,878)		
	*n*	HR	*p, p*_FDR_	*n*	HR	*p, p*_FDR_	*n*	HR	*p, p*_FDR_	*n*	HR	*p, p*_FDR_
All causes	483			2,185			1.265			564		
Age and sex		0.97 [0.88, 1.07]	.558, .710		0.97 [0.94, 1.02]	.292, .508		0.89 [0.83, 0.94]	< .001, < .001		0.83 [0.76, 0.90]	< .001, < .001
All covariates		0.97 [0.88, 1.07]	.521, .695		0.96 [0.92, 1.01]	.068, .169		0.89 [0.83, 0.94]	< .001, < .001		0.86 [0.79, 0.94]	.001, .004
Cancer	340			1,497			758			317		
Age and sex		0.94 [0.84, 1.06]	.319, .507		0.96 [0.91, 1.01]	.101, .212		0.87 [0.80, 0.94]	< .001, < .001		0.73 [0.65, 0.82]	< .001, < .001
All covariates		0.94 [0.84, 1.06]	.313, .507		0.94 [0.89, 1.00]	.001, .001		0.87 [0.81, 0.94]	< .001, < .001		0.80 [0.71, 0.90]	< .001, < .001
Cardiovascular disease	75			402			313			135		
Age and sex		0.99 [0.78, 1.27]	.960, .985		0.94 [0.84, 1.04]	.222, .404		0.87 [0.77, 0.98]	.022, .068		0.87 [0.73, 1.03]	.107, .214
All covariates		1.00 [0.78, 1.28]	.987, .986		0.91 [0.82, 1.01]	.072, .169		0.86 [0.77, 0.90]	.016, .049		0.85 [0.71, 1.02]	.077, .171
Respiratory disease	56			257			247			128		
Age and sex		0.96 [0.72, 1.28]	.789, .852		0.95 [0.83, 1.08]	.451, .644		0.81 [0.71, 0.93]	.003, .011		0.93 [0.78, 1.11]	.414, .613
All covariates		0.91 [0.68, 1.21]	.511, .695		0.92 [0.81, 1.05]	.222, .404		0.79 [0.68, 0.90]	.001, .004		0.91 [0.76, 1.09]	.330, .508
External causes	54			205			103			60		
Age and sex		0.98 [0.74, 1.30]	.879, .925		1.20 [1.05, 1.27]	.008, .027		0.95 [0.78, 1.17]	.646, .760		0.95 [0.73, 1.23]	.684, .782
All covariates		0.96 [0.72, 1.07]	.760, .844		1.17 [1.02, 1.33]	.027, .072		0.95 [0.77, 1.16]	.606, .735		0.93 [0.71, 1.20]	.568, .710

Note: Effect estimates were first adjusted for age and sex only and then further adjusted for other covariates at baseline: health behaviors (smoking status, frequency of alcohol intake, number of types of exercise engaged in, and daily consumption of fruits and vegetables), physical attributes (body mass index, forced expiratory volume in 1 s, systolic blood pressure, and grip strength), reaction time, existing illness (diagnosis of vascular or heart problems, diabetes, cancer, asthma, chronic lung disease, deep vein thrombosis, or pulmonary embolism at baseline), and socioeconomic position (Townsend index score and highest educational qualification). Values inside brackets are 95% confidence intervals. *p* = uncorrected *p* value; *p*_FDR_ = *p* value corrected for the false discovery rate.

We examined the extent to which higher neuroticism might compensate for the adverse influence of poor self-rated health on mortality by comparing the main effect of poor self-rated health and the effect of its interaction with neuroticism. In the case of all-cause mortality, after adjustment for all the covariates, poor self-rated health was associated with a more than threefold increase in risk of death, HR = 3.27, 95% CI = [2.84, 3.77]; including the interaction of poor self-rated health with neuroticism in the model reduced this risk only slightly, HR = 2.99, 95% CI = [2.28, 4.04]. In the case of cancer-related mortality, poor self-rated health was also associated with a more than threefold increase in risk of death, HR = 3.26, 95% CI = [2.74, 3.89]; including the interaction of poor self-rated health with neuroticism in the model reduced the risk a little more than in the case of all-cause mortality, but again, the reduction in risk was small, HR = 2.77, 95% CI = [2.35, 3.27].

In summary, exploratory analyses suggested that the relationships between neuroticism and mortality from all causes and cancer, but not those between neuroticism and mortality from cardiovascular disease, respiratory disease, or external causes, varied by level of self-rated health. Higher neuroticism was protective against mortality from all causes and from cancer only in participants who rated their health as fair or poor. After FDR correction, higher neuroticism remained significantly associated with reduced risk of death from cancer in participants who rated their health as fair or poor, but was associated with reduced risk of death from all causes only in those who rated their health as fair. Comparison of the main effect of poor self-rated health with the effect of its interaction with neuroticism on mortality risk suggested that higher neuroticism reduced risk of death from all causes and from cancer in participants with poor self-rated health by only a small amount.

### Neuroticism facets and mortality

At the suggestion of a referee, we explored the apparent protective association between neuroticism and mortality that was revealed after adjustment for self-rated health. The full exploratory structural equation model of the neuroticism items from the EPQ-R Short Form is presented in the Supplemental Material available online. This structure consisted of a general Neuroticism factor, onto which all items loaded; two facets were orthogonal to the general Neuroticism factor and correlated with each other at .312, *p* < .0001. The general Neuroticism factor correlated .96 with score on the full neuroticism scale. The three items with the highest loadings on the first facet, which we labeled “anxious-tense,” were “Would you call yourself a nervous person?” (loading = .608), “Do you suffer from ‘nerves’?” (loading = .490), and “Would you call yourself tense or ‘highly strung’?” (loading = .352). The four items with the highest loadings on the second facet, which we labeled “worried-vulnerable,” were “Do you worry too long after an embarrassing experience?” (loading = .568), “Are your feelings easily hurt?” (loading = .399), “Are you ever troubled by feelings of guilt?” (loading = .315), and “Are you a worrier?” (loading = .309). The factor determinacies for the general factor and two facets were .919, .790, and .721, respectively. For the factor scores extracted from this analysis, the anxious-tense facet and the worried-vulnerable facet correlated .26 and .38, respectively, with scores on the full neuroticism scale, both *p*s < .0001. Scores on these facets correlated .07 and .12, respectively, with scores on the general factor, and .43 with each other, all *p*s < .0001.

Table 4 shows the associations between a 1-*SD* increment in each facet and all-cause and cause-specific mortality, when the facets were entered simultaneously along with the general Neuroticism factor. The anxious-tense facet was not significantly associated with risk of all-cause or cause-specific mortality. Higher scores on the worried-vulnerable facet were associated with a significantly reduced risk of death from all causes in the age- and sex-adjusted analysis, HR = 0.88, 95% CI = [0.86, 0.91]. After further adjustment for all the covariates, the effect was attenuated but remained significant, even after FDR correction, HR = 0.94, 95% CI = [0.90, 0.97]. In the age- and sex-adjusted models, higher worried-vulnerable scores were also associated with a significantly reduced risk of death from cancer, HR = 0.93, 95% CI = [0.89, 0.97]; cardiovascular disease, HR = 0.84, 95% CI = [0.78, 0.91]; and respiratory disease, HR = 0.84, 95% CI = [0.77, 0.91], but not from external causes. However, none of these associations remained significant after adjustment for all the covariates and correction for multiple testing.

**Table 4.** Hazard Ratios (HRs) for All-Cause and Cause-Specific Mortality per 1-*SD* Increment in the Anxious-Tense and Worried-Vulnerable Facets of Neuroticism, Examined Simultaneously (*N* = 321,456)

Cause of death and adjustments	Anxious-tense facet		Worried-vulnerable facet	
	HR	*p, p*_FDR_	HR	*p, p*_FDR_
All causes (*n* = 4,497)				
Age, sex, and Neuroticism factor	1.00 [0.98, 1.03]	.905, .937	0.88 [0.86, 0.91]	< .001, < .001
All covariates	0.99 [0.96, 1.03]	.652, .819	0.94 [0.90, 0.97]	< .001, < .001
Cancer (*n* = 2,912)				
Age, sex, and Neuroticism factor	0.96 [0.92, 1.00]	.065, .180	0.93 [0.89, 0.97]	< .001, < .001
All covariates	0.96 [0.92, 1.00]	.072, .180	0.97 [0.92, 1.01]	.097, .194
Cardiovascular disease (*n* = 925)				
Age, sex, and Neuroticism factor	1.00 [0.93, 1.07]	.920, .935	0.84 [0.78, 0.91]	< .001, < .001
All covariates	0.99 [0.92, 1.06]	.708, .833	0.93 [0.86, 1.00]	.045, .150
Respiratory disease (*n* = 688)				
Age, sex, and Neuroticism factor	1.02 [0.94, 1.11]	.588, .819	0.84 [0.77, 0.91]	< .001, < .001
All covariates	0.98 [0.90, 1.06]	.581, .819	0.93 [0.85, 1.01]	.081, .180
External causes (*n* = 422)				
Age, sex, and Neuroticism factor	1.03 [0.90, 1.13]	.655, .819	0.92 [0.82, 1.02]	.114, .207
All covariates	1.00 [0.90, 1.10]	.937, .937	0.97 [0.87, 1.08]	.631, .819

Note: Effect estimates were first adjusted for age, sex, and the general Neuroticism factor and then further adjusted for other covariates at baseline: health behaviors (smoking status, frequency of alcohol intake, number of types of exercise engaged in, and daily consumption of fruits and vegetables), physical attributes (body mass index, forced expiratory volume in 1 s, systolic blood pressure, and grip strength), reaction time, existing illness (diagnosis of vascular or heart problems, diabetes, cancer, asthma, chronic lung disease, deep vein thrombosis, or pulmonary embolism at baseline), socioeconomic position (Townsend index score and highest educational qualification), and self-rated health. Values inside brackets are 95% confidence intervals. *p* = uncorrected *p* value; *p*_FDR_ = *p* value corrected for the false discovery rate.

### The role of health behaviors

To explore whether physical activity, fruit and vegetable consumption, smoking, or alcohol use might help explain the protective effect of higher neuroticism on mortality from all causes and cancer in participants with fair or poor self-rated health, we examined whether the correlations between neuroticism and these health behaviors differed between participants who rated their health as fair or poor and those who rated their health as excellent or good. Before the data were stratified by self-rated health, after adjustments for age and sex, higher neuroticism was modestly but significantly correlated (*p* < .0001) with less healthy behaviors: It was negatively correlated with eating at least five portions of fruits and vegetables daily (*r* = −.042) and with the number of types of physical activity that participants engaged in (*r* = −.100), but positively correlated with being a current smoker (*r* = .050) and drinking alcohol daily or nearly daily (*r* = .015). Comparing the corresponding correlations and their 95% CIs between participants who rated their health as excellent or good and those who rated their health as fair or poor showed that there was no significant difference between these groups of participants in any of these behaviors. Moreover, in multivariable models of all-cause and cancer-related mortality, the effect sizes for neuroticism were essentially the same whether or not we included these health-behavior covariates. Thus, our results suggest that these behaviors do not account for the association between higher neuroticism and lower mortality risk in people with fair or poor self-rated health.

We explored the relationship between neuroticism and health behaviors further by investigating whether the presence of disease at baseline (diagnosis of vascular or heart problems, diabetes, cancer, asthma, chronic lung disease, deep vein thrombosis, or pulmonary embolism) moderated this relationship. Age- and sex-adjusted correlations between neuroticism and the health behaviors were very similar in participants with and without diagnosed disease, and analyses showed that they did not differ significantly between these two groups.

### The role of diagnosed disease

Participants who had a diagnosed disease at baseline were more likely to rate their health as fair or poor, compared with those who did not have such a diagnosis, and they were also more likely to have died during the follow-up period. We examined whether having any diagnosis participants were asked about at baseline (i.e., vascular or heart problems, diabetes, cancer, asthma, chronic lung disease, deep vein thrombosis, or pulmonary embolism) moderated the associations between neuroticism and all-cause and cancer-related mortality in participants who viewed their health as fair or poor. The *p* values for the interaction terms were not statistically significant (*p*s = .749 and .942, respectively).

### The effect of missing covariate data

The analyses described thus far were based on 321,456 participants (64% of the 502,655 people recruited to UK Biobank) who had complete data on neuroticism, self-rated health, and all the other covariates at baseline. To explore whether excluding people with missing covariate data biased our findings, we carried out a sensitivity analysis including the 401,265 people who had data on neuroticism and self-rated health. The associations were similar to those described in the previous sections. For example, the age- and sex-adjusted HR for death from all causes per 1-*SD* increment in neuroticism was 1.10, 95% CI = [1.08, 1.13]; after further adjustment for self-rated health, the HR changed to 0.94, 95% CI = [0.91, 0.96]. In our sample of participants with complete data on all variables, the corresponding HRs were 1.06, 95% CI = [1.03, 1.09], and 0.93, 95% CI = [0.90, 0.96], respectively.

## Discussion

In this prospective study, age- and sex-adjusted analyses showed that higher neuroticism was associated with a slight increase in mortality risk overall. However, after adjustment for other covariates, and, in particular, self-rated health, higher neuroticism was associated with reduced mortality from all causes, cancer, cardiovascular disease, and respiratory disease, but not external causes. The relationships between neuroticism and mortality from all causes and cancer varied according to self-rated health. Tests of the overall interaction between neuroticism and self-rated health did not meet our imposed criterion for significance (*p* < .001) in the case of mortality from all causes (*p* = .003) and were significant in the case of mortality from cancer (*p* = .0007). Exploratory analyses in which we stratified the sample by self-rated health showed that higher neuroticism was associated with reduced mortality from all causes and from cancer in participants who rated their health as fair or poor; only the association with cancer was significant after FDR correction. The compensatory effect of higher neuroticism on risk of death from all causes or cancer in participants with poor self-rated health was small.

We also examined whether two Neuroticism facets—anxious-tense and worried-vulnerable—that were independent of the common Neuroticism variance were associated with mortality. Higher scores on the worried-vulnerable facet were associated with a reduced risk of death from all causes. This effect persisted after adjustment for all the covariates and survived correction for multiple testing. Higher scores on the worried-vulnerable facet were also associated with lower mortality from cancer, cardiovascular disease, and respiratory disease, but only in the age- and sex-adjusted models. The anxious-tense facet was not associated with mortality.

Although higher neuroticism has been linked with poorer subjective health (Goodwin & Engstrom, 2002; Watson & Pennebaker, 1989), it might be protective against death if it leads individuals to be vigilant in taking care of their health (Friedman, 2000). We found some support for that notion: Among people who rated their health as poor or fair, higher neuroticism was associated with a reduced mortality from all causes and cancer. No such effect was observed in participants with excellent self-rated health. We found no indication to suggest that diet, exercise, smoking, or drinking explained the association between neuroticism and mortality in participants with fair or poor self-rated health, but our data were restricted to behavior at the start of the study, and so may not reflect changes in these behaviors made subsequently. Higher neuroticism was associated with poorer health behaviors, although the size of all these correlations was small. We found no evidence that these correlations differed between participants with excellent or good self-rated health and participants with fair or poor self-rated health. There was also no evidence that these correlations differed between participants who had diagnosed disease at baseline and those who did not. If concerns about health underlie our finding that higher neuroticism is linked with lower mortality from all causes and cancer in people with relatively poor self-rated health, concerns about health do not appear to be manifested via the health behaviors we examined at baseline.

There is evidence that higher neuroticism is associated with greater use of health-care services (Cuijpers et al., 2010). This propensity to seek medical help in response to worries about health could plausibly result in earlier identification of cancer, and greater likelihood of survival. We were unable to investigate whether the protective effect of higher neuroticism in people with fair or poor self-rated health was due to seeking professional advice in response to symptoms or compliance with medical treatment, but our finding that higher neuroticism among these participants was associated with a reduction in risk of death from cancer is consistent with that explanation, as is our observation that higher scores on the worried-vulnerable facet of the Neuroticism factor were associated with reduced mortality from all causes. It is worth noting that higher scores on this facet were associated with lower mortality even when we did not adjust for self-rated health.

Strengths of our study include the number of deaths in our large sample and the inclusion of data on a range of potential confounding factors. One limitation is that no data were available on personality traits other than neuroticism. We could not examine whether conscientiousness, for example, moderated neuroticism’s relationship with mortality. Being high in conscientiousness may lead individuals who are high in neuroticism to live a particularly healthy lifestyle, possibly in response to health concerns (Vollrath & Torgersen, 2002; Weston & Jackson, 2015). Weston and Jackson (2015) found that after the onset of chronic physical disease, people who were high in neuroticism and high in conscientiousness, “healthy neurotics,” smoked less. This combination of neuroticism and conscientiousness was negatively associated with smoking only after disease onset. Weston and Jackson therefore suggested that high conscientiousness may enable individuals high in neuroticism to act on their anxiety by making behavioral changes when they are confronted by disease. We found no evidence that the relationship between neuroticism and health behaviors differed between participants with and without physical illness at baseline, but were unable to examine the potential impact of conscientiousness on this relationship.

Another limitation of our study is that our follow-up period was relatively short—on average, 6.25 years. We cannot gauge whether the association between higher neuroticism and reduced mortality in people with poor self-rated health persists over longer periods. A final limitation of this study is that the analyses concerning the interaction of neuroticism and self-rated health were exploratory, as we found a significant interaction effect only in the case of mortality from cancer. The lack of significant interaction effects is probably attributable to a combination of our very conservative criterion for significance and the fact that the power to detect interaction effects is considerably lower than that to detect main effects (McClelland & Judd, 1993). Researchers should thus repeat our analyses in other data sets, and use an alpha criterion that better balances power to detect interaction effects and avoidance of a high Type I error rate.

The findings of this study raise the question of why neuroticism becomes protective against mortality from all causes and cancer in people with fair or poor self-rated health. These protective effects were not explained by the health behaviors we assessed (smoking, exercise, fruit and vegetable intake and alcohol consumption) and did not vary according to the presence of diagnosed disease. It may be that individuals with higher neuroticism are more vigilant about their health if they perceive it to be less than excellent. They may be more aware of bodily, including autonomic, symptoms and may be more likely to consult their doctor, perhaps thereby increasing the likelihood of earlier diagnosis and prompt treatment. As we noted earlier, our findings regarding the Neuroticism facets provide some evidence in support of this idea: The lower risk of all-cause mortality seen in individuals with high scores on the worried-vulnerable facet could have been due to a greater propensity to seek medical advice. Future analysis of primary-care records for this cohort—not currently available—could lend further support to this explanation. If prompt seeking of medical advice is indeed a mechanism underlying the covert protective effect of neuroticism, researchers may need to reevaluate the evidence regarding the economic costs of neuroticism in terms of use of health-care resources (Cuijpers et al., 2010).

The present results suggest that perhaps the most promising avenue for future research would be a closer examination of the role of Neuroticism’s facets, for example, the six—anxiety, angry-hostility, depression, self-consciousness, impulsiveness, and vulnerability—operationalized by the Revised NEO Personality Inventory (Costa & McCrae, 1992). A study of the association between “nuances” (Mõttus, Kandler, Bleidorn, Riemann, & McCrae, 2017) of neuroticism (e.g., the 48 items from the Revised NEO Personality Inventory Neuroticism scale) no doubt will also yield insights into when and why neuroticism might harm or protect health.

## Supplementary Material

Supplementary material

## References

[bibr1-0956797617709813] Almada S. J. Zonderman A. B. Shekelle R. B. Dyer A. R. Daviglus M. L. Costa P. T. Jr. Stamler J. (1991). Neuroticism and cynicism and risk of death in middle-aged men: The Western Electric Study. Psychosomatic Medicine, 53, 165–175. 2031070 10.1097/00006842-199103000-00006

[bibr2-0956797617709813] Batty G. D. Der G. Macintyre S. Deary I. J. (2006). Does IQ explain socioeconomic inequalities in health? Evidence from a population based cohort study in the west of Scotland. British Medical Journal, 332, 580–584. doi:10.1136/bmj.38723.660637.AE 16452104 PMC1397779

[bibr3-0956797617709813] Benjamini Y. Drai D. Elmer G. Kafkafi N. Golani I. (2001). Controlling the false discovery rate in behavior genetics research. Behavioural Brain Research, 125, 279–284. doi:10.1016/S0166-4328(01)00297-2 11682119

[bibr4-0956797617709813] Benyamini Y. Idler E. L. (1999). Community studies reporting association between self-rated health and mortality: Additional studies, 1995 to 1998. Research on Aging, 21, 392–401. doi:10.1177/0164027599213002

[bibr5-0956797617709813] Chapman B. Duberstein P. Lyness J. M. (2007). Personality traits, education, and health-related quality of life among older adult primary care patients. Journals of Gerontology: Series B, 62, 343–352. doi:10.1093/geronb/62.6.P343 18079419

[bibr6-0956797617709813] Costa P. T. Jr. McCrae R. R. (1987). Neuroticism, somatic complaints, and disease: Is the bark worse than the bite? Journal of Personality, 55, 299–316. doi:10.1111/j.1467-6494.1987.tb00438.x 3612472

[bibr7-0956797617709813] Costa P. T. Jr. McCrae R. R. (1992). Revised NEO Personality Inventory (NEO-PI-R) and NEO Five-Factor Inventory (NEO-FFI): Professional manual. Odessa, FL: Psychological Assessment Resources.

[bibr8-0956797617709813] Costa P. T. Jr. McCrae R. R. (1995). Domains and facets: Hierarchical personality assessment using the Revised NEO Personality Inventory. Journal of Personality Assessment, 64, 21–50. doi:10.1207/s15327752jpa6401_2 16367732

[bibr9-0956797617709813] Costa P. T. Jr. Weiss A. Duberstein P. R. Friedman B. Siegler I. C. (2014). Personality facets and all-cause mortality among Medicare patients aged 66 to 102 years: A follow-on study of Weiss and Costa (2005). Psychosomatic Medicine, 76, 370–378. doi:10.1097/PSY.0000000000000070 24933014 PMC4096682

[bibr10-0956797617709813] Cuijpers P. Smit F. Penninx B. W. de Graaf R. ten Have M. Beekman A. T. (2010). Economic costs of neuroticism: A population-based study. Archives of General Psychiatry, 67, 1086–1093. doi:10.1001/archgenpsychiatry.2010.130 20921124

[bibr11-0956797617709813] Deary I. J. Der G. Ford G. (2001). Reaction times and intelligence differences: A population-based cohort study. Intelligence, 29, 389–399. doi:10.1016/S0160-2896(01)00062-9

[bibr12-0956797617709813] Eysenck S. B. G. Eysenck H. J. Barrett P. (1985). A revised version of the Psychoticism scale. Personality and Individual Differences, 6, 21–29. doi:10.1016/0191-8869(85)90026-1

[bibr13-0956797617709813] Friedman H. S. (2000). Long-term relations of personality and health: Dynamisms, mechanisms, tropisms. Journal of Personality, 68, 1089–1107. doi:10.1111/1467-6494.00127 11130733

[bibr14-0956797617709813] Friedman H. S. Kern M. L. Reynolds C. A. (2010). Personality and health, subjective well-being, and longevity. Journal of Personality, 78, 179–215. doi:10.1111/j.1467-6494.2009.00613.x 20433617

[bibr15-0956797617709813] Gale C. R. Batty G. D. Osborn D. P. Tynelius P. Whitley E. Rasmussen F. (2012). Association of mental disorders in early adulthood and later psychiatric hospital admissions and mortality in a cohort study of more than 1 million men. Archives of General Psychiatry, 69, 823–831. doi:10.1001/archgenpsychiatry.2011.2000 22868936 PMC4170756

[bibr16-0956797617709813] Ganna A. Ingelsson E. (2015). 5 year mortality predictors in 498,103 UK Biobank participants: A prospective population-based study. The Lancet, 386, 533–540. doi:10.1016/S0140-6736(15)60175-1 26049253

[bibr17-0956797617709813] Goodwin R. Engstrom G. (2002). Personality and the perception of health in the general population. Psychological Medicine, 32, 325–332. doi:10.1017/S0033291701005104 11866326

[bibr18-0956797617709813] Gow A. J. Whiteman M. C. Pattie A. Deary I. J. (2005). Goldberg’s ‘IPIP’ Big-Five factor markers: Internal consistency and concurrent validation in Scotland. Personality and Individual Differences, 39, 317–329. doi:10.1016/j.paid.2005.01.011

[bibr19-0956797617709813] Idler E. L. Benyamini Y. (1997). Self-rated health and mortality: A review of twenty-seven community studies. Journal of Health and Social Behavior, 38, 21–37. doi:10.2307/2955359 9097506

[bibr20-0956797617709813] Iwasa H. Masui Y. Gondo Y. Inagaki H. Kawaai C. Suzuki T. (2008). Personality and all-cause mortality among older adults dwelling in a Japanese community: A five-year population-based prospective cohort study. American Journal of Geriatric Psychiatry, 16, 399–405. doi:10.1097/JGP.0b013e3181662ac9 18403571

[bibr21-0956797617709813] Jennrich R. I. Bentler P. M. (2011). Exploratory bi-factor analysis. Psychometrika, 76, 537–549. doi:10.1007/s11336-011-9218-4 22232562 PMC3253271

[bibr22-0956797617709813] Jennrich R. I. Bentler P. M. (2012). Exploratory bi-factor analysis: The oblique case. Psychometrika, 77, 442–454. doi:10.1007/s11336-012-9269-1 27519775

[bibr23-0956797617709813] Jokela M. Batty G. D. Nyberg S. T. Virtanen M. Nabi H. Singh-Manoux A. Kivimaki M. (2013). Personality and all-cause mortality: Individual-participant meta-analysis of 3,947 deaths in 76,150 adults. American Journal of Epidemiology, 178, 667–675. doi:10.1093/aje/kwt170 23911610 PMC3755650

[bibr24-0956797617709813] Jorm A. F. Christensen H. Henderson S. Korten A. E. Mackinnon A. J. Scott R. (1993). Neuroticism and self-reported health in an elderly community sample. Personality and Individual Differences, 15, 515–521. doi:10.1016/0191-8869(93)90334-Y

[bibr25-0956797617709813] Korten A. E. Jorm A. F. Jiao Z. Letenneur L. Jacomb P. A. Henderson A. S.. . . Rogers B. (1999). Health, cognitive, and psychosocial factors as predictors of mortality in an elderly community sample. Journal of Epidemiology & Community Health, 53, 83–88. doi:10.1136/jech.53.2.83 10396468 PMC1756829

[bibr26-0956797617709813] Kotov R. Gamez W. Schmidt F. Watson D. (2010). Linking “big” personality traits to anxiety, depressive, and substance use disorders: A meta-analysis. Psychological Bulletin, 136, 768–821. doi:10.1037/a0020327 20804236

[bibr27-0956797617709813] Löckenhoff C. E. Terracciano A. Ferrucci L. Costa P. T. Jr. (2012). Five-factor personality traits and age trajectories of self-rated health: The role of question framing. Journal of Personality, 80, 375–401. doi:10.1111/j.1467-6494.2011.00724.x 21299558 PMC3248623

[bibr28-0956797617709813] Lonnqvist J. E. Verkasalo M. Haukka J. Nyman K. Tiihonen J. Laaksonen I.. . . Henriksson M. (2009). Premorbid personality factors in schizophrenia and bipolar disorder: Results from a large cohort study of male conscripts. Journal of Abnormal Psychology, 118, 418–423. doi:10.1037/a0015127 19413416

[bibr29-0956797617709813] Malouff J. M. Thorsteinsson E. B. Schutte N. S. (2005). The relationship between the five-factor model of personality and symptoms of clinical disorders: A meta-analysis. Journal of Psychopathology and Behavioral Assessment, 27, 101–114. doi:10.1007/s10862-005-5384-y

[bibr30-0956797617709813] McClelland G. H. Judd C. M. (1993). Statistical difficulties of detecting interactions and moderator effects. Psychological Bulletin, 114, 376–390. doi:10.1037/0033-2909.114.2.376 8416037

[bibr31-0956797617709813] Mõttus R. Kandler C. Bleidorn W. Riemann R. McCrae R. R. (2017). Personality traits below facets: The consensual validity, longitudinal stability, heritability, and utility of personality nuances. Journal of Personality and Social Psychology, 112, 474–490. doi:10.1037/pspp0000100 27124378

[bibr32-0956797617709813] Muthén L. K. Muthén B. O. (1998–2015). Mplus user’s guide (7th ed.). Los Angeles, CA: Author.

[bibr33-0956797617709813] Neeleman J. Bijl R. Ormel J. (2004). Neuroticism, a central link between somatic and psychiatric morbidity: Path analysis of prospective data. Psychological Medicine, 34, 521–531. doi:10.1017/S0033291703001193 15259837

[bibr34-0956797617709813] Ploubidis G. B. Grundy E. (2009). Personality and all cause mortality: Evidence for indirect links. Personality and Individual Differences, 47, 203–208. doi:10.1016/j.paid.2009.02.022

[bibr35-0956797617709813] Russ T. C. Hamer M. Stamatakis E. Starr J. M. Batty G. D. Kivimaki M. (2012). Association between psychological distress and mortality: Individual participant pooled analysis of 10 prospective cohort studies. British Medical Journal, 345, Article e4933. doi:10.1136/bmj.e4933 PMC3409083 22849956

[bibr36-0956797617709813] Shipley B. A. Weiss A. Der G. Taylor M. D. Deary I. J. (2007). Neuroticism, extraversion, and mortality in the UK Health and Lifestyle Survey: A 21-year prospective cohort study. Psychosomatic Medicine, 69, 923–931. doi:10.1097/PSY.0b013e31815abf83 17991814

[bibr37-0956797617709813] Sudlow C. Gallacher J. Allen N. Beral V. Burton P. Danesh J.. . . Collins R. (2015). UK Biobank: An open access resource for identifying the causes of a wide range of complex diseases of middle and old age. PLoS Medicine, 12, Article e1001779. doi:10.1371/journal.pmed.1001779 PMC4380465 25826379

[bibr38-0956797617709813] Townsend P. Phillimore P. Beattie A. (1988). Health and deprivation: Inequality and the North. Beckenham, England: Croom Helm.

[bibr39-0956797617709813] Turiano N. A. Mroczek D. K. Moynihan J. Chapman B. P. (2013). Big 5 personality traits and interleukin-6: Evidence for “healthy Neuroticism” in a US population sample. Brain, Behavior, and Immunity, 28, 83–89. doi:10.1016/j.bbi.2012.10.020 PMC3545072 23123863

[bibr40-0956797617709813] Vollrath M. Torgersen S. (2002). Who takes health risks? A probe into eight personality types. Personality and Individual Differences, 32, 1185–1197. doi:10.1016/S0191-8869(01)00080-0

[bibr41-0956797617709813] Watson D. Pennebaker J. W. (1989). Health complaints, stress, and distress: Exploring the central role of negative affectivity. Psychological Review, 96, 234–254. doi:10.1037/0033-295X.96.2.234 2710874

[bibr42-0956797617709813] Weiss A. Costa P. T. Jr. (2005). Domain and facet personality predictors of all-cause mortality among Medicare patients aged 65 to 100. Psychosomatic Medicine, 67, 724–733. doi:10.1097/01.psy.0000181272.58103.18 16204430

[bibr43-0956797617709813] Weiss A. Gale C. R. Batty G. D. Deary I. J. (2009). Emotionally stable, intelligent men live longer: The Vietnam Experience Study cohort. Psychosomatic Medicine, 71, 385–394. doi:10.1097/PSY.0b013e318198de78 19251871

[bibr44-0956797617709813] Weiss A. Gale C. R. Batty G. D. Deary I. J. (2013). A questionnaire-wide association study of personality and mortality: The Vietnam Experience Study. Journal of Psychosomatic Research, 74, 523–529. doi:10.1016/j.jpsychores.2013.02.010 23731751 PMC3697823

[bibr45-0956797617709813] Weston S. J. Jackson J. J. (2015). Identification of the healthy neurotic: Personality traits predict smoking after disease onset. Journal of Research in Personality, 54, 61–69. doi:10.1016/j.jrp.2014.04.008

[bibr46-0956797617709813] World Health Organisation. (1992). International Statistical Classification of Diseases and Related Health Problems, 10th Revision (ICD-10). Geneva, Switzerland: Author.

