# Supplementary material for: When Is Higher Neuroticism Protective Against Death? Findings From UK Biobank
Source: Psychol Sci. 2017 Jul 13;28(9):1345–57. doi: 10.1177/0956797617709813 (PMC5595241; doi:10.1177/0956797617709813)
Supplement: Supplementary material [file Gale_Supplemental_Material.pdf]

```
Mplus VERSION 7.4 (Linux)
MUTHEN & MUTHEN
03/16/2017  11:03 AM

INPUT INSTRUCTIONS

  TITLE: Bi-factor model
  DATA: FILE = "data for bifactor model.dat";
  VARIABLE:

  NAMES = eid mood miserable irritable hurtflings fedup nervous worrier tense
       embarassed nerves lonely guilt reactiontime townsend_score smokestatus
       selfrated_health BMI dead survivaltime age_assessment sex diabetes_diag
       vasculardis_diag numbertypes_exercise alcohol fiveplus_fruitveg fev1
maxgrip
       sysbp highest_quals cancer_diag cvd_anymention cancer_anymention
       respiratory_anymention external_anymention DVT chronicbronchitis asthma
       lungclot complete_data;

  USEVARIABLES = eid mood miserable irritable hurtflings fedup nervous worrier
tense
        embarassed nerves lonely guilt;

  IDVARIABLE = eid;

  MISSING=.;

  ANALYSIS:

  ROTATION = BI-GEOMIN;

  MODEL:

  fg f1 f2 BY mood miserable irritable hurtflings fedup nervous worrier tense
         embarassed nerves lonely guilt (*1);

  OUTPUT:

  STDY;

  FSDETERMINACY;

  SAVEDATA:
  FILE IS ukbb_bifactor.sav;
  save is fscores;
  format is free;




*** WARNING in VARIABLE command
  Note that only the first 8 characters of variable names are used in the
output.
  Shorten variable names to avoid any confusion.
   1 WARNING(S) FOUND IN THE INPUT INSTRUCTIONS



Bi-factor model

SUMMARY OF ANALYSIS

Number of groups                                                 1
Number of observations                                      321456
```

```
Number of dependent variables                                   12
Number of independent variables                                  0
Number of continuous latent variables                            3

Observed dependent variables

  Continuous
   MOOD        MISERABLE   IRRITABLE   HURTFLINGS  FEDUP       NERVOUS
   WORRIER     TENSE       EMBARASSED  NERVES      LONELY      GUILT

Continuous latent variables

  EFA factors
  *1:   FG          F1          F2

Variables with special functions

  ID variable           EID

Estimator                                                       ML
Rotation                                                 BI-GEOMIN
Row standardization                                    CORRELATION
Type of rotation                                           OBLIQUE
Information matrix                                        OBSERVED
Maximum number of iterations                                  1000
Convergence criterion                                    0.500D-04
Maximum number of steepest descent iterations                   20
Maximum number of iterations for H1                           2000
Convergence criterion for H1                             0.100D-03
Optimization Specifications for the Exploratory Factor Analysis
Rotation Algorithm
  Number of random starts                                       30
  Maximum number of iterations                               10000
  Derivative convergence criterion                       0.100D-04

Input data file(s)
  data for bifactor model.dat

Input data format  FREE


SUMMARY OF DATA

     Number of missing data patterns             1


COVARIANCE COVERAGE OF DATA

Minimum covariance coverage value   0.100


     PROPORTION OF DATA PRESENT


           Covariance Coverage
```

	MOOD	MISERABL	IRRITABL	HURTFLIN	FEDUP
MOOD	1.000				
MISERABL	1.000	1.000			
IRRITABL	1.000	1.000	1.000		
HURTFLIN	1.000	1.000	1.000	1.000	
FEDUP	1.000	1.000	1.000	1.000	1.000
NERVOUS	1.000	1.000	1.000	1.000	1.000

WORRIER	1.000	1.000	1.000	1.000	1.000
TENSE	1.000	1.000	1.000	1.000	1.000
EMBARASS	1.000	1.000	1.000	1.000	1.000
NERVES	1.000	1.000	1.000	1.000	1.000
LONELY	1.000	1.000	1.000	1.000	1.000
GUILT	1.000	1.000	1.000	1.000	1.000

Covariance Coverage

	NERVOUS	WORRIER	TENSE	EMBARASS	NERVES
NERVOUS	1.000				
WORRIER	1.000	1.000			
TENSE	1.000	1.000	1.000		
EMBARASS	1.000	1.000	1.000	1.000	
NERVES	1.000	1.000	1.000	1.000	1.000
LONELY	1.000	1.000	1.000	1.000	1.000
GUILT	1.000	1.000	1.000	1.000	1.000

Covariance Coverage

	LONELY	GUILT
LONELY	1.000	
GUILT	1.000	1.000

THE MODEL ESTIMATION TERMINATED NORMALLY

MODEL FIT INFORMATION

Number of Free Parameters 57

Loglikelihood

H0 Value	-1911155.114	
H1 Value	-1899006.494	

Information Criteria

Akaike (AIC)	3822424.229	
Bayesian (BIC)	3823033.024	
Sample-Size Adjusted BIC (n* = (n + 2) / 24)	3822851.875	

Chi-Square Test of Model Fit

Value	24297.240	
Degrees of Freedom	33	
P-Value	0.0000	

RMSEA (Root Mean Square Error Of Approximation)

Estimate	0.048	
90 Percent C.I.	0.047	0.048
Probability RMSEA <= .05	1.000	

CFI/TLI

CFI	0.975	
TLI	0.950	

Chi-Square Test of Model Fit for the Baseline Model

Value	979346.753
Degrees of Freedom	66
P-Value	0.0000

SRMR (Standardized Root Mean Square Residual)

Value	0.019

MODEL RESULTS

	Estimate	S.E.	Est./S.E.	Two-Tailed P-Value
**FG BY**				
MOOD	0.364	0.001	322.885	0.000
MISERABLE	0.331	0.001	294.753	0.000
IRRITABLE	0.219	0.001	268.895	0.000
HURTFLINGS	0.228	0.001	228.148	0.000
FEDUP	0.344	0.001	293.282	0.000
NERVOUS	0.191	0.003	69.986	0.000
WORRIER	0.240	0.002	148.234	0.000
TENSE	0.179	0.002	116.795	0.000
EMBARASSED	0.201	0.001	154.099	0.000
NERVES	0.176	0.002	80.843	0.000
LONELY	0.170	0.001	245.178	0.000
GUILT	0.202	0.001	229.102	0.000
**F1 BY**				
MOOD	-0.064	0.004	-14.948	0.000
MISERABLE	-0.076	0.004	-21.273	0.000
IRRITABLE	0.012	0.002	4.911	0.000
HURTFLINGS	-0.012	0.001	-10.885	0.000
FEDUP	-0.077	0.004	-19.630	0.000
NERVOUS	0.251	0.002	108.809	0.000
WORRIER	0.081	0.002	50.574	0.000
TENSE	0.131	0.002	65.871	0.000
EMBARASSED	0.004	0.000	14.399	0.000
NERVES	0.197	0.002	96.972	0.000
LONELY	-0.010	0.002	-6.306	0.000
GUILT	-0.006	0.001	-10.094	0.000
**F2 BY**				
MOOD	-0.023	0.001	-17.205	0.000
MISERABLE	0.013	0.001	14.050	0.000
IRRITABLE	-0.019	0.001	-16.398	0.000
HURTFLINGS	0.199	0.001	136.711	0.000
FEDUP	-0.009	0.001	-11.587	0.000
NERVOUS	0.011	0.001	16.016	0.000
WORRIER	0.154	0.001	112.900	0.000
TENSE	-0.007	0.001	-10.026	0.000
EMBARASSED	0.283	0.002	181.932	0.000
NERVES	-0.011	0.001	-14.762	0.000
LONELY	0.021	0.001	22.139	0.000
GUILT	0.142	0.001	112.991	0.000
**F1 WITH**				
FG	0.000	0.000	95.646	0.000
**F2 WITH**				

FG	0.000	0.000	-66.442	0.000
F1	0.312	0.008	40.453	0.000
**Intercepts**				
MOOD	0.426	0.001	488.148	0.000
MISERABLE	0.412	0.001	474.788	0.000
IRRITABLE	0.272	0.001	346.795	0.000
HURTFLINGS	0.535	0.001	608.024	0.000
FEDUP	0.382	0.001	445.733	0.000
NERVOUS	0.219	0.001	299.875	0.000
WORRIER	0.536	0.001	609.024	0.000
TENSE	0.165	0.001	252.425	0.000
EMBARASSED	0.463	0.001	526.606	0.000
NERVES	0.201	0.001	284.769	0.000
LONELY	0.171	0.001	257.573	0.000
GUILT	0.280	0.001	353.270	0.000
**Variances**				
FG	1.000	0.000	999.000	999.000
F1	1.000	0.000	999.000	999.000
F2	1.000	0.000	999.000	999.000
**Residual Variances**				
MOOD	0.106	0.000	247.667	0.000
MISERABLE	0.127	0.000	307.659	0.000
IRRITABLE	0.150	0.000	368.650	0.000
HURTFLINGS	0.158	0.001	295.419	0.000
FEDUP	0.111	0.000	267.275	0.000
NERVOUS	0.069	0.000	154.974	0.000
WORRIER	0.153	0.000	330.102	0.000
TENSE	0.090	0.000	325.094	0.000
EMBARASSED	0.127	0.001	170.136	0.000
NERVES	0.093	0.000	263.206	0.000
LONELY	0.112	0.000	380.938	0.000
GUILT	0.141	0.000	345.693	0.000

STANDARDIZED MODEL RESULTS

STDY Standardization

	Estimate	S.E.	Est./S.E.	Two-Tailed P-Value
**FG BY**				
MOOD	0.736	0.002	389.669	0.000
MISERABLE	0.673	0.002	348.670	0.000
IRRITABLE	0.492	0.002	317.648	0.000
HURTFLINGS	0.458	0.002	256.925	0.000
FEDUP	0.708	0.002	343.150	0.000
NERVOUS	0.463	0.007	70.813	0.000
WORRIER	0.481	0.003	155.944	0.000
TENSE	0.481	0.004	120.288	0.000
EMBARASSED	0.403	0.002	162.721	0.000
NERVES	0.439	0.005	82.047	0.000
LONELY	0.452	0.002	281.747	0.000
GUILT	0.450	0.002	258.293	0.000
**F1 BY**				
MOOD	-0.130	0.009	-14.948	0.000
MISERABLE	-0.154	0.007	-21.279	0.000
IRRITABLE	0.027	0.006	4.911	0.000
HURTFLINGS	-0.024	0.002	-10.886	0.000

FEDUP	-0.158	0.008	-19.633	0.000
NERVOUS	0.608	0.006	109.962	0.000
WORRIER	0.161	0.003	50.666	0.000
TENSE	0.352	0.005	66.209	0.000
EMBARASSED	0.009	0.001	14.404	0.000
NERVES	0.490	0.005	98.078	0.000
LONELY	-0.027	0.004	-6.307	0.000
GUILT	-0.014	0.001	-10.095	0.000
**F2 BY**				
MOOD	-0.047	0.003	-17.208	0.000
MISERABLE	0.027	0.002	14.050	0.000
IRRITABLE	-0.043	0.003	-16.405	0.000
HURTFLINGS	0.399	0.003	140.441	0.000
FEDUP	-0.018	0.002	-11.588	0.000
NERVOUS	0.026	0.002	16.016	0.000
WORRIER	0.309	0.003	114.679	0.000
TENSE	-0.020	0.002	-10.027	0.000
EMBARASSED	0.568	0.003	190.125	0.000
NERVES	-0.028	0.002	-14.763	0.000
LONELY	0.057	0.003	22.156	0.000
GUILT	0.315	0.003	115.248	0.000
**F1 WITH**				
FG	0.000	0.000	95.646	0.000
**F2 WITH**				
FG	0.000	0.000	-66.442	0.000
F1	0.312	0.008	40.453	0.000
**Intercepts**				
MOOD	0.861	0.002	416.956	0.000
MISERABLE	0.837	0.002	408.537	0.000
IRRITABLE	0.612	0.002	318.299	0.000
HURTFLINGS	1.072	0.002	484.480	0.000
FEDUP	0.786	0.002	389.584	0.000
NERVOUS	0.529	0.002	280.875	0.000
WORRIER	1.074	0.002	484.985	0.000
TENSE	0.445	0.002	240.776	0.000
EMBARASSED	0.929	0.002	440.164	0.000
NERVES	0.502	0.002	268.347	0.000
LONELY	0.454	0.002	245.230	0.000
GUILT	0.623	0.002	323.283	0.000
**Variances**				
FG	1.000	0.000	999.000	999.000
F1	1.000	0.000	999.000	999.000
F2	1.000	0.000	999.000	999.000
**Residual Variances**				
MOOD	0.435	0.002	241.727	0.000
MISERABLE	0.525	0.002	312.305	0.000
IRRITABLE	0.756	0.002	489.558	0.000
HURTFLINGS	0.637	0.002	320.145	0.000
FEDUP	0.471	0.002	263.921	0.000
NERVOUS	0.406	0.003	152.841	0.000
WORRIER	0.616	0.002	359.565	0.000
TENSE	0.649	0.002	362.545	0.000
EMBARASSED	0.512	0.003	170.504	0.000
NERVES	0.575	0.002	272.180	0.000
LONELY	0.793	0.001	555.227	0.000
GUILT	0.700	0.002	412.597	0.000

R-SQUARE

Observed Variable	Estimate	S.E.	Est./S.E.	Two-Tailed P-Value
MOOD	0.565	0.002	314.201	0.000
MISERABL	0.475	0.002	282.479	0.000
IRRITABL	0.244	0.002	157.989	0.000
HURTFLIN	0.363	0.002	182.641	0.000
FEDUP	0.529	0.002	296.155	0.000
NERVOUS	0.594	0.003	223.670	0.000
WORRIER	0.384	0.002	224.236	0.000
TENSE	0.351	0.002	196.098	0.000
EMBARASS	0.488	0.003	162.542	0.000
NERVES	0.425	0.002	200.975	0.000
LONELY	0.207	0.001	144.836	0.000
GUILT	0.300	0.002	176.483	0.000

QUALITY OF NUMERICAL RESULTS

Condition Number for the Information Matrix 0.587E-04
(ratio of smallest to largest eigenvalue)

SUMMARY OF FACTOR SCORES

FACTOR SCORE INFORMATION (COMPLETE-DATA PATTERN)

FACTOR DETERMINACIES

FG	0.919
F1	0.790
F2	0.721

SAMPLE STATISTICS FOR ESTIMATED FACTOR SCORES

SAMPLE STATISTICS

Means

	FG	FG_SE	F1	F1_SE	F2
1	0.000	0.394	0.000	0.613	0.000

Means

	F2_SE
1	0.693

Covariances

	FG	FG_SE	F1	F1_SE	F2
FG	0.845				
FG_SE	0.000	0.000			
F1	0.051	0.000	0.624		
F1_SE	0.000	0.000	0.000	0.000	
F2	0.080	0.000	0.245	0.000	0.520

```
 F2_SE          0.000         0.000         0.000         0.000         0.000


           Covariances
              F2_SE
              ________
 F2_SE          0.000


           Correlations
              FG            FG_SE         F1            F1_SE         F2
              ________      ________      ________      ________      ________
 FG             1.000
 FG_SE        999.000         1.000
 F1             0.070       999.000         1.000
 F1_SE        999.000       999.000       999.000         1.000
 F2             0.121       999.000         0.430       999.000         1.000
 F2_SE        999.000       999.000       999.000       999.000       999.000


           Correlations
              F2_SE
              ________
 F2_SE          1.000


SAVEDATA INFORMATION


  Save file
    ukbb_bifactor.sav

  Order of variables

    MOOD
    MISERABL
    IRRITABL
    HURTFLIN
    FEDUP
    NERVOUS
    WORRIER
    TENSE
    EMBARASS
    NERVES
    LONELY
    GUILT
    EID
    FG
    FG_SE
    F1
    F1_SE
    F2
    F2_SE

  Save file format           Free

  Save file record length    10000


     Beginning Time:  11:03:29
        Ending Time:  11:03:45
       Elapsed Time:  00:00:16
```

MUTHEN & MUTHEN
3463 Stoner Ave.
Los Angeles, CA 90066

Tel: (310) 391-9971
Fax: (310) 391-8971
Web: www.StatModel.com
Support: Support@StatModel.com

Copyright (c) 1998-2015 Muthen & Muthen
